# Diagnostic Accuracy of a SARS-CoV-2 Antigen Test Obtained by Mid-turbinate Nasal Swabs

**DOI:** 10.7759/cureus.87120

**Published:** 2025-07-01

**Authors:** John W Epling, Matthew B Lowery, Alexandra A Weir, Martha M Tenzer, Tonja M Locklear, Anthony W Baffoe-Bonnie, Paul R Skolnik

**Affiliations:** 1 Family and Community Medicine, Virginia Tech Carilion School of Medicine, Roanoke, USA; 2 Family and Community Medicine, Carilion Clinic, Roanoke, USA; 3 Medicine, University of North Carolina, Chapel Hill, USA; 4 Health Analytics and Research, Carilion Clinic, Roanoke, USA; 5 Infectious Diseases, Virginia Tech Carilion School of Medicine, Roanoke, USA; 6 Basic Science Education, Virginia Tech Carilion School of Medicine, Roanoke, USA

**Keywords:** antigen, covid-19, diagnostic test accuracy, mid-turbinate nasal swab, outpatient management, sars-cov-2

## Abstract

Context

In the early months of the COVID-19 pandemic, polymerase chain reaction (PCR) testing for SARS-CoV-2 was difficult to obtain and took several days to return a result. Our health system wished to explore the use of the Quidel Sofia (Quidel Corporation, San Diego, CA) antigen test to diagnose COVID-19 in our primary care clinics, but the test was approved for emergency use authorization by the US Food and Drug Administration (FDA) with only 250 test subjects. In addition, because it was important to avoid aerosol-generating procedures in primary care clinics, it was necessary to test the diagnostic performance of the antigen test using mid-turbinate (MT) swabs rather than the approved nasopharyngeal (NP) swab technique.

Objective

The aim of this study was to assess the diagnostic test characteristics of a SARS-CoV-2 antigen test performed using MT nasal swabs compared with the presumed reference standard PCR test by NP swab.

Study design

This was a prospective cohort study.

Population studied

Adult outpatients with symptoms consistent with mild to moderate COVID-19. We attempted to recruit 800 subjects to provide statistical assurance that the test sensitivity was at least 90%.

Intervention/instrument

After informed consent, subjects underwent MT nasal swabbing for antigen testing, followed by NP swabbing for PCR testing.

Outcome measures

These included sensitivity, specificity, positive and negative predictive values, and likelihood ratios, all with associated 95% confidence intervals.

Results

Due to recruitment difficulty (subject reluctance and staffing issues at the testing centers), we recruited only 117 subjects. Sensitivity was 0.750 (95% CI 0.566, 0.885), and specificity was 0.988 (95% CI 0.936, 1.000). Positive predictive value was 0.960 (95% CI 0.796, 0.999), and negative predictive value was 0.913 (95% CI 0.836, 0.962). The likelihood ratio for a positive test was 63.75 (95% CI 8.99, 451.97), and the likelihood ratio for a negative test was 0.25 (95% CI 0.14, 0.46).

Conclusions

This antigen test for SARS-CoV-2 was of reasonable clinical utility to rule in COVID-19 in a low-prevalence environment, but concerns about high-risk populations and the ramifications of false negatives limited its use. Difficulty recruiting subjects and the resultant delay in the results made it impossible to implement this antigen testing in primary care practices, but it is hoped that these data will contribute to the accumulation of evidence about diagnostic testing for COVID-19.

## Introduction

Testing for SARS-CoV-2-related disease was an important and complex topic during the most active months of the COVID-19 pandemic. There were a variety of testing solutions available, released for clinical use under the US Food and Drug Administration Emergency Use Authorization (EUA). This authorization was granted with a minimal amount of research to demonstrate the accuracy of these tests, which left questions about how the tests would perform in different clinical contexts. Of particular concern was testing of symptomatic patients, such as healthcare workers, where accuracy was important and time-sensitive. The need for more rapid-turnaround testing solutions was growing daily as commercial and public health laboratories faced processing delays for the gold standard real-time polymerase chain reaction (RT-PCR) assays [1,2]. At the time, nasopharyngeal (NP) swabs, the primary specimen collection method for RT-PCR, were believed to pose a risk of aerosol generation [3,4]. As a result, their use necessitated not only higher-level personal protective equipment but also performance in rooms with enhanced ventilation or extended downtime between patients to allow for adequate air exchange. As a result, there was an interest in developing testing methods in primary care offices and other practices that did not require aerosol-generating procedures.

Antigen-based testing, increasingly available from commercial labs, promised a more rapid result, often within 15 minutes, but at the cost of a lower sensitivity compared to PCR testing. The Sofia antigen test (Quidel Corporation, San Diego, CA) was, at the time of this study, one of a few antigen tests approved by the FDA with an EUA [5]. The FDA’s EUA letter for the antigen test stated that “Negative results should be treated as presumptive and confirmed with a molecular assay, if necessary, for patient management” [6,7]. As our institution faced a higher demand for rapid testing of its healthcare workforce, we needed information about how well this antigen test could rule out COVID-19 in symptomatic healthcare workers.

The primary objective of this study was to compare the diagnostic performance of the Quidel Sofia SARS-CoV-2 antigen test obtained by mid-turbinate (MT) nasal swab with the reference standard RT-PCR testing from NP swabs in a population of adult patients with symptoms consistent with COVID-19 disease. The secondary objective was to calculate additional clinically oriented statistics (predictive values and likelihood ratios from the diagnostic test performance statistics, especially the negative predictive value) to understand the value of this test to rule out disease in symptomatic subjects. This study was previously presented as a research abstract at the 49^th^ Annual Meeting of the North American Primary Care Research Group (NAPCRG) from November 19-21, 2021.

## Materials and methods

We designed a cross-sectional diagnostic testing study to be performed at two outpatient COVID-19 testing centers that our health system had deployed for employees as well as for the broader community. We recruited adults (>=18 years) with symptoms of COVID-19 infection for fewer than five days. Both the character of the symptoms and the duration of the symptoms were adjudicated by the clinicians ordering the testing, and, as part of the order, the clinicians were asked to verify that the symptoms met Centers for Disease Control (CDC) criteria for COVID-19 [8], as well as the duration of the symptoms. These subjects, which included but were not limited to healthcare workers from our system, were referred to the testing centers for same-day testing by RT-PCR testing using an NP swab. The referral centers were conveniently located in two locations in our health system footprint and were used for outpatient testing only.

The test of interest was an MT nasal swab of both nares using the Sofia rapid antigen test [7]. The reference standard used was one of two RT-PCR tests (one developed by Quest Laboratories Inc. (Secaucus, NJ), and the other developed through a collaboration between our affiliated research institute and the state department of health [9]) using an NP swab from both nares. The two RT-PCR tests probe for the same standard genetic sequence and were considered equivalent. The two testing labs were used interchangeably in our region to overcome issues of availability of test processing for the southwest Virginia region. The antigen test was collected prior to the RT-PCR test to reduce the chance that the detection would be improved due to any rhinorrhea produced by NP swabbing. Testing staff at the centers were trained in the informed consent process by our health system research staff and in specimen collection by their clinical managers according to the manufacturers’ specifications for both the antigen and RT-PCR tests. The study was self-funded by Carilion Clinic, Roanoke, VA, and there was no involvement of the manufacturer of the antigen test. The study was reviewed and approved by the Carilion Clinic Institutional Review Board for the Protection of Human Subjects (#IRB-20-1088) and is registered at clinicaltrials.gov (NCT04610489).

Data collection and management

As the nature of this study was to determine overall operational characteristics of the antigen test, there were no demographic or illness data collected beyond ensuring that the subjects met the inclusion criteria as outlined above. The antigen testing samples were processed immediately on-site at the regional testing centers, and the results and identifiers were entered into a Research Electronic Data Capture (REDCap) secure database (Vanderbilt University, Nashville, TN) for the study. The RT-PCR test results were processed at Quest Laboratories or at the Fralin Biomedical Research Institute, Roanoke, VA, in collaboration with the Virginia Department of Health. The testing staff was not blinded, but both tests were run on computerized analyzers, usually in batches, and the PCR testing was not contingent on the result of the antigen test. Results of the RT-PCR tests were sent to the patient’s ordering physician and were extracted from the electronic health record for entry into the REDCap database by study personnel.

Sample size determination

Because of operational concerns for healthcare workers, it was determined that the lowest acceptable sensitivity of the antigen test (relative to the RT-PCR) was 90%, and thus, we hypothesized that the antigen test would demonstrate at least 90% sensitivity. The average test-positive rate in the testing centers for all symptomatic patients for the month prior to the study was approximately 10%. A sample size of 800 subjects using a 5% threshold for significance was calculated to achieve 80% power to detect a difference of 0.099 between two diagnostic tests whose sensitivities would be 0.999 and 0.900.

Analysis

The results of the testing were analyzed using R (v. 3.6, 2019, with the EpiR package (R Development Core Team, R Foundation for Statistical Computing, Vienna, Austria)). The following descriptive statistics were calculated for the antigen test (relative to the reference standard RT-PCR test): prevalence, sensitivity, specificity, positive and negative predictive values, likelihood ratios for positive and negative tests, and overall test accuracy. Confidence intervals (95%) for these results were calculated using the Wilson score method.

## Results

There were 117 subjects recruited for the study despite extending the recruitment period by several months. Recruitment was challenged by multiple staffing changes and instability at the sites (unrelated to the study) and general reluctance of potential subjects to have another swab performed. Both antigen and RT-PCR testing results were available for all subjects (Table 1). The antigen test was shown to have a sensitivity of 75.0% and a specificity of 98.8%. The antigen test results produced a robust likelihood ratio for a positive test of 63.75 but only a “fair” likelihood ratio for a negative test of 0.25. The overall accuracy of the antigen test was 92.4% (Table 2).

**Table 1:** 2x2 table of COVD-19 testing results Antigen: Quidel Sofia SARS-CoV-2 antigen test; RT-PCR: real-time polymerase chain reaction test

Antigen test	RT-PCR positive	RT-PCR negative
Antigen positive	24	1
Antigen negative	8	84

**Table 2:** Diagnostic test characteristics of Quidel Sofia antigen tests

Test characteristic	Point estimate	95% Confidence interval
Sensitivity	0.75	0.566, 0.885
Specificity	0.988	0.936, 1.000
Positive predictive value	0.96	0.796, 0.999
Negative predictive value	0.913	0.836, 0.962
Likelihood ratio: positive test	63.75	8.99, 451.97
Likelihood ratio – negative test	0.25	0.14, 0.46
Diagnostic accuracy	0.924	0.859, 0.964

## Discussion

The Quidel Sofia antigen test can effectively rule in COVID-19 infection when positive, but cannot rule it out in a symptomatic outpatient adult population within five days of symptom onset. This inability to rule out COVID-19 made it unsuitable for our use in healthcare worker testing and of limited utility for other adult testing. False negatives could result in greater numbers of healthcare-associated infections (amongst healthcare workers as well as infection of patients) and in missed opportunities for early isolation and treatment, especially for high-risk individuals. As a result, RT-PCR testing was implemented as standard in our testing centers and outpatient clinics, despite the often-long wait times for results.

Quidel Corporation's package insert for the Sofia SARS Antigen test listed a 96.7% sensitivity and 100% specificity in the data for 209 symptomatic subjects submitted for FDA evaluation [7]. Systematic reviews from mid-2020 included early evaluations of the performance of antigen testing (from other manufacturers) in symptomatic subjects and reported sensitivities between 56 and 86% [10, 11], which was more consistent with the results of our study.

Multiple studies and systematic reviews that included the Quidel Sofia antigen test have been conducted since this study was completed in 2021. Most of these studies were conducted to determine the specificity and sensitivity of COVID-19 antigen tests and to compare the performance of various antigen tests. These studies included both symptomatic and asymptomatic patients. Some studies also included patients who were being screened for work suitability or were part of active surveillance programs. The types of tests that were used in these studies included but were not limited to lateral flow immunoassays (LFIA), chemiluminescence immunoassays (CLIA), enzyme-linked fluorescent assays (ELFA), enzyme-linked immunosorbent assays (ELISA), fluorescence immunoassays (FIA), immunochromatographic assays (ICG), antigen rapid diagnostic tests (Ag-RDT), rapid antigen detection (RAD) tests, and rapid antigen tests.

As summarized in the more recent relevant systematic reviews, the sensitivities of the tests used in these studies ranged from 34.8% to 82%, with most of the sensitivities in the 70s, and the specificities ranged from 97.8% to 100%, with most in the high 90s [12-17]. The CDC continues to recommend backup testing for negative COVID-19 antigen testing, either by serially repeating the tests or by using RT-PCR testing [18]. The availability of increasingly rapid RT-PCR testing has challenged assumptions of the utility of rapid antigen testing in the clinical environment [19].

There were multiple limitations to this study that affected the results and analysis. Recruitment of the initially planned sample size was prevented by the reluctance of the potential subjects and by multiple workforce changes in the study personnel due to the ad hoc nature of staffing during the more intense period of the COVID-19 pandemic. The wide confidence intervals for our diagnostic performance statistics are a consequence of this low sample size. Additional clinical and demographic data that could have enhanced the analysis was not collected because of the pragmatic scope of our research question, so specific applicability questions could not be addressed; however, it should be noted that these results would not apply to patients who are asymptomatic or who have had more than five days of COVID-19-like symptoms. The testing site served both the community and the healthcare organization’s employees, so the sample may not reflect true population-based results. In addition, there were two different RT-PCR testing modalities used for our reference standard because of the limited capacity of PCR testing at that time, which is a validity concern for diagnostic testing studies. Therefore, while the results of this study are consistent with subsequent data concerning antigen testing, all of these issues limit the validity and generalizability of this study’s data.

## Conclusions

The Quidel Sofia COVID-19 antigen test had good ability to rule in COVID-19 disease when positive, but our study could not confirm its ability to rule out COVID-19 in a recently symptomatic adult outpatient population. Our limited results are consistent with subsequent research on these tests.

The availability of point-of-care RT-PCR testing helped our institution manage sick employee management and pre-procedural screening for patients. The use of the quicker reference standard test to detect COVID-19 helps reduce the transmission of the virus in our hospitals and ambulatory practices.
